# Using naltrexone to validate a human laboratory test system to screen new medications for alcoholism (TESMA)- a randomized clinical trial

**DOI:** 10.1038/s41398-023-02404-7

**Published:** 2023-04-05

**Authors:** Maik Spreer, Xina Grählert, Ina-Maria Klut, Feras Al Hamdan, Wolfgang H. Sommer, Martin H. Plawecki, Sean O’Connor, Michael Böttcher, Cathrin Sauer, Michael N. Smolka, Ulrich S. Zimmermann

**Affiliations:** 1 Department of Psychiatry and Psychotherapy, University Hospital Carl Gustav Carus of the Technische Universität Dresden, Dresden, Germany; 2 Coordination Centre for Clinical Trials, Faculty of Medicine Carl Gustav Carus, Technische Universität Dresden, Dresden, Germany; 3 Hospital-Pharmacy, University Hospital Carl Gustav Carus Dresden, Dresden, Germany; 4 Institute of Psychopharmacology, Central Institute of Mental Health, Medical Faculty Mannheim, University of Heidelberg, Mannheim, Germany; 5 Bethanian Hospital for Psychiatry, Psychosomatics and Psychotherapy Greifswald, Greifswald, Germany; 6 Department of Psychiatry, Indiana University School of Medicine, Indianapolis, IN USA; 7 Department of Toxicology, MVZ Medizinische Labore Dessau Kassel GmbH, Dessau-Rosslau, Germany; 8 Department of Addiction Medicine and Psychotherapy, kbo Isar-Amper-Klinikum Region München, Munich, Germany

**Keywords:** Clinical pharmacology, Addiction, Human behaviour, Pharmacodynamics

## Abstract

This registered clinical trial sought to validate a laboratory test system devised to screen medications for alcoholism treatment (TESMA) under different contingencies of alcohol reinforcement. Forty-six nondependent, but at least medium-risk drinkers were given the opportunity to earn intravenous infusions of ethanol, or saline, as rewards for work in a progressive-ratio paradigm. Work demand pattern and alcohol exposure dynamics were devised to achieve a gradual shift from low-demand work for alcohol (WFA) permitting quickly increasing breath alcohol concentrations (BrAC) to high-demand WFA, which could only decelerate an inevitable decrease of the previously earned BrAC. Thereby, the reward contingency changed, modeling different drinking motivations. The experiment was repeated after at least 7 days of randomized, double-blinded treatment with naltrexone, escalated to 50 mg/d, or placebo. Subjects treated with naltrexone reduced their cumulative WFA (cWFA) slightly more than participants receiving placebo. This difference was not statistically significant in the preplanned analysis of the entire 150 min of self-administration, i.e., our primary endpoint (*p* = 0.471, Cohen’s *d* = 0.215). Naltrexone serum levels correlated with change in cWFA (*r* = −0.53; *p* = 0.014). Separate exploratory analyses revealed that naltrexone significantly reduced WFA during the first, but not the second half of the experiment (Cohen’s *d* = 0.643 and 0.14, respectively). Phase-dependent associations of WFA with changes in subjective stimulation, wellbeing and desire for alcohol suggested that the predominant reinforcement of WFA was positive during the first phase only, and might have been negative during the second. We conclude that the TESMA is a safe and practical method. It bears the potential to quickly and efficiently screen new drugs for their efficacy to attenuate positively reinforced alcohol consumption. It possibly also provides a condition of negative reinforcement, and for the first time provides experimental evidence suggesting that naltrexone’s effect might depend on reward contingency.

## Introduction

Alcohol dependence as defined by ICD-10 is a frequent and costly psychiatric illness causing significant medical and social harm [GBD 2016 Alcohol Collaborators [1]]. Worldwide, there are six medications approved to prevent relapse or reduce drinking: disulfiram (inhibition of acetaldehyde dehydrogenase), naltrexone and nalmefene (opiate receptor antagonists), acamprosate (putative modulation of the glutamatergic system), gamma-hydroxybutyrate (modulation of GABA and GHB receptors), and baclofen (GABA-B receptor agonist). Their effect sizes are generally considered to be small-to-medium [2] with the possible exception of supervised disulfiram [3]. More effective medications are badly needed, but many compounds which attenuated addiction-like behaviors in preclinical models failed in expensive clinical trials with alcohol-dependent patients [4]. Some progress has been made since Litten et al. [5] described the overall goals for medications development several years ago, and accumulating evidence suggests that e.g. topiramate, gabapentin, varenicline and ondansetron might also help to improve alcohol-related outcomes [6].

In order to bridge the gap between preclinical research and the clinical treatment of alcoholism, several human laboratory models have been suggested [7] as methods to discriminate which compounds warrant a human clinical trial. Among them, experimental alcohol self-administration (ASA) appears particularly promising, since this approach was able to predict that naltrexone, nalmefene and varenicline do, while acamprosate and rimonabant do not, reduce alcohol consumption in alcohol-dependent patients [7, 8].

ASA experiments can be tailored to investigate specific aspects of alcohol consumption and addiction. Free-access paradigms, for example, measure alcohol “liking” [9], a construct which can be observed in nondependent social drinkers. The requirement to perform work in order to get access to alcohol [10–12], rather relates to aspects of alcohol “wanting” which, according to the incentive salience theory, represents a pathological drinking motivation due to previous sensitization of the reward system to alcohol [13].

The standard method of ASA is oral ingestion; easy to perform and conforming best with the behavior to be altered. However, disadvantages with oral ASA are that (i) it is impossible to standardize the extent and time course of brain alcohol exposure, (ii) attempts to blind the taste and smell of alcohol, and the amount already consumed, have been largely unsatisfactory, (iii) due to the inevitable alcohol reservoir in the stomach, complying with safety limits is only possible by restricting the number and/or time course of possible drinks, limiting peak breath alcohol concentrations (BrACs) achieved, and thus narrowing bandwidth of the main outcome variable. Intravenous (i.v.) ethanol self-administration avoids these drawbacks and allows for faster, fully-controlled alcohol exposure dynamics, both when BrAC is ascending (during a reward) and descending (in between rewards) [14]. Subjective responses to alcohol have been shown to be highly correlated and non-differentiable between oral and i.v. administration when an individual’s exposure profile is similar [15].

Building on these insights, we devised a new laboratory paradigm, the Test system to Screen Medications for Alcoholism (TESMA). The TESMA paradigm covers several of the above-described aspects of addiction-related behaviors. Our approach was to investigate the willingness to perform voluntary work for “rewards” (VWR); either an incremental i.v. infused exposure ethanol that quickly increased BrAC by the same fixed amount within and across subjects, or of the infusate vehicle, i.e., saline. The latter served as a control condition, to check whether subjects worked specifically for alcohol or for nonspecific reasons such as boredom or curiosity. In between alcohol rewards, the infusion rate is designed to produce a steep linear decline of BrAC, faster than after oral ingestion, potentially increasing the contrast between the subjective perceptions of feeling vs. not feeling a reward and emphasizing the subjective effects of falling BrACs. A progressive-ratio work schedule was implemented; requiring an increasing number of iterations of a task for each successive work-set before a reward was delivered. In a pilot study, this method was able to detect medication effects on operant alcohol self-administration [11].

As a result of this setup, the contingency of reinforcement changed over experimental time course within each session. The first few work-sets could be finished quickly, rendering the workload so low that it approximated free-access ASA and allowed for rapidly increasing BrACs. In this first experimental (“ascending”) phase, work for alcohol (WFA) was predominantly positively reinforced. However, if subjects continued steady WFA, the time required to complete each work-set exponentially increased, and—in between rewards—the BrAC continued its pre-determined descent. Towards the end of the experiment, work for the next reward took so long that, during work time, the BrAC dropped by more than the following reward increased it, marking the beginning of a phase of “futility”. In this second experimental (“plateau”) phase, BrAC inevitably decreased, but the rate of descent could still be slowed down by continuous WFA. We assume that an unintended drop of BrAC is perceived aversive, therefore WFA during the plateau phase can be seen as negatively rather than positively reinforced (See Fig. S4 in the supplement for graphical description of an example).

As a second consequence, the continuous transition from low- to high-demand operant ASA in subjects working continuously for alcohol also implies that the incentive components driving WFA might shift over experimental time. In the beginning, WFA is so easy that it comes very close to a free-access paradigm, where ASA is driven by both hedonic (“liking”) and motivational (“wanting”) aspects of alcohol consumption. However, with increasing work demand and decreasing positive reinforcement, the participant’s behavior towards the end of the experiment might be more determined by motivation.

The aim of the present study was to validate the TESMA paradigm by detecting an effect of naltrexone, a drug which not only helps alcohol-dependent patients to drink less [16], but also reduces laboratory oral ASA in dependent and nondependent drinkers [17]. Our hypothesis was that naltrexone, compared to placebo, would reduce the willingness to work for alcohol, operationalized as cumulative WFA (cWFA), over the entire experimental course of time. All study procedures (Clinical Trials NCT02652585, EudraCT number 2015-002831-16) were reviewed and approved by the ethics committee at the Technische Universität Dresden and fully complied with the Declaration of Helsinki and Good Clinical Practice. This paper reports the primary outcome and some secondary endpoints. Other secondary outcomes were already reported [18, 19].

## Materials and methods

### Participants

**Fig. 1:**
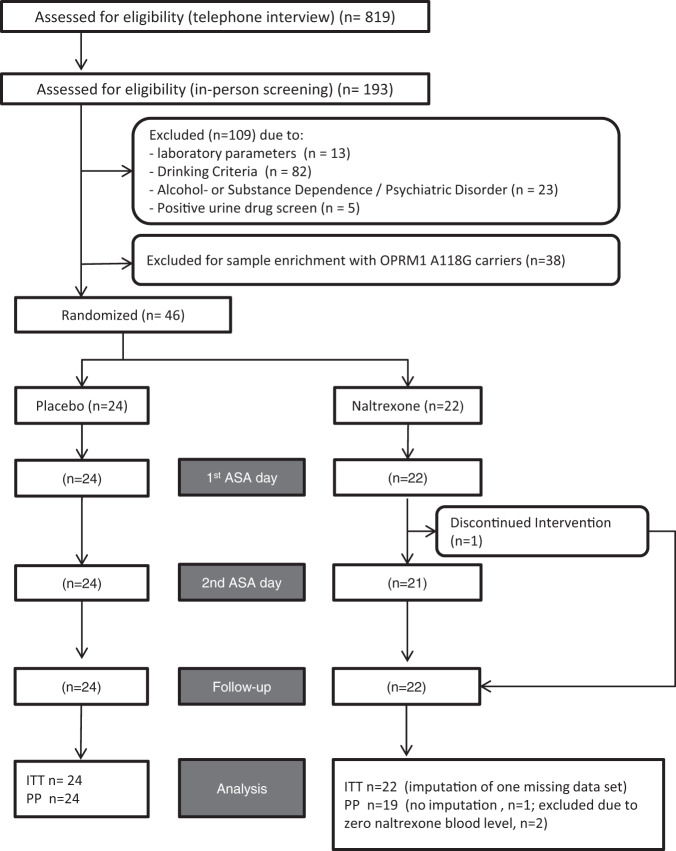
Recruitment and retention flowchart. CONSORT flowchart of recruitment and retention throughout the study. ITT intention-to treat, PP per-protocol.

All subjects were recruited by local advertisements (newspapers, social media and flyer distribution in bars and restaurants). We included healthy male and female non-treatment-seeking volunteers aged 25–55 years who, during the last 45 days before screening, (i) were at least medium-risk drinkers (WHO: men at least 41 g, women at least 31 g alcohol per day), (ii) had at least one binge day per week with >100 g ethanol for men and >75 g for women, and (iii) had at least four non-consecutive alcohol abstinent days in the preceding 45 days. Key exclusion criteria were a history or presence of alcohol withdrawal symptoms (CIWA-Ar-Score >5), any prior alcohol-related treatment, current ICD-10 alcohol- or substance dependence, except for nicotine; current use of illicit or other psychotropic drugs including opiates, any psychiatric disorder requiring current treatment, routine laboratory parameters suggesting relevant liver or pancreas injury, anemia, renal insufficiency, or the body weight of >130 kg. Figure 1 describes recruitment and retention of the participants.

Sample size calculation can be found in the supplement. Eligible subjects provided informed consent and underwent in-person screening, comprising BrAC measurement, general health screening with brief physical examination, blood sampling for blood count, liver, renal, and pancreas functions and electrolytes, demographic information, a 45-day Timeline Follow-Back interview [20], lifetime drinking history, alcohol craving (OCDS-Score), and family history of alcoholism [21]. Urine was tested for pregnancy (Abbott Diagnostics Medical Co., Ltd. hCG COMBO) and illicit drugs (Nal von minden, Moers, Germany; see supplement for substances tested). All subjects were genotyped for the OPRM1 A118G polymorphism (rs1799971), which has an allele frequency of ~15% in the Caucasian population in Germany [22] and is the best validated functional variant of the mu-opioid receptor with established effects on the rewarding properties of alcohol [23] and the effects of naltrexone [24]. Since they may respond better to naltrexone [25–27], we enriched the sample population with G-allele carriers by allowing only three out of each block of six subsequently screened subjects to participate. They were selected by first including all GG subjects, second all heterozygous G-allele carriers and third the AA subjects of each block, according to their sequence of giving informed consent, respectively.

### General experimental methods

The laboratory setting and general experimental process was as described in [28]. Subjects undertook a progressive-ratio ASA session (session 1), where they performed work to earn alcohol or saline infusions as a reward. They could decide whether to perform work for either alcohol or saline, or to refrain from doing so, at their discretion. Subjects were instructed that their session goal was to produce pleasant alcohol effects like they usually would when drinking at a party, but to avoid unpleasant alcohol effects. Subjects were then randomized to receive either naltrexone (25 mg in the morning for 3 days, then 50 mg in the morning until 28 days total) or placebo in a double-blind, placebo-controlled parallel-group design, stratified by sex and genotype. After at least 7 (7–16; mean 8.2 ± 1.9) days on medication, the experiment was repeated (session 2). Thereafter, subjects continued drug treatment for a total of 28 days and during that time underwent two more experiments (sessions 3 and 4) with fMRI scanning during experimenter-controlled alcohol administration [18]. A follow-up visit to ensure safety was performed 3–5 days after discontinuing study medication.

### Alcohol/saline infusion procedures

We used the Computer-Assisted Alcohol Infusion System (CAIS [9, 11];) to administer ethanol and saline. CAIS software calculates the infusion rates necessary to control BrAC. Ethanol was administered as a 6.0% (v/v) solution in 0.9% saline at a maximum rate of 1998 ml/hr. Each session began with a priming interval, during which subjects were first prompted to work for the “water” reward two times, and then for the alcohol reward four times. This procedure served to practice the work task, demonstrate the sensations associated with each reward, and provide a priming alcohol exposure. Each alcohol reward increased BrAC by 13 mg/dL within 3.0 min and was followed by a linear decline of BrAC by −0.8 mg/dL per minute until the next reward or as long as was possible pharmacokinetically. Requests for “water” triggered an infusion of normal saline, administered at a rate of up to 5 ml/min for 3 min via the same i.v. line. The priming procedure raised BrAC to 40.1 ± 5.2 mg/dL within ~18 min. A waiting time until minute 25 ensued, during which BrAC fell to 34.8 ± 4.6 mg/dL. Thereafter, the actual voluntary work for reward (VWR) phase began, with analytical time defined as T = 0 and lasting for 150 min. Subjects knew that they were discharged from the lab not before 3.5 h after the infusion ended. BrAC readings were obtained approximately every 20 min using an Alcotest 6810 med device (Draeger Sicherheitstechnik, Lübeck, Germany) to ensure safety and to refine CAIS infusion rate calculations. Concerning code availability, the CAIS computer code is delivered to investigators upon agreement with the authors (SOC and MHP). It comprises a compiled file and is not available for modification in the interest of safety. A password-protected setup file is part of each CAIS paradigm; parameters affecting the paradigm’s function can be changed by the investigator.

### Subjective responses

About 100 mm visual analog scale measures of alcohol-induced stimulation, sedation, negative effects, well-being, guessed the number of “drinks” received, drunkenness, thirst, and “desire” were obtained. To assess the latter, we asked whether subjects would “like to have some more alcohol now”. We suggest that term “desire” should not be confused with the pathological alcohol “craving” occurring in alcohol-dependent patients, noting that our participants were nondependent risky drinkers. Subjective measures were obtained at baseline, during the priming interval (minute 15), during the ascending phase (minute 75), and at the end of both ASA sessions using visual analog scales (0–100).

### Work task

A constant attention task (CAT) was employed as work as described in [11, 29]. In brief, subjects initiated a CAT trial by pressing and holding a button, then quickly releasing it upon a visual prompt. Iterations of this trial took 7.3 s on average. CAIS automatically adjusted the reaction time required to successfully complete a trial to compensate for any alcohol-induced impairment, fatigue, or individual differences in responsiveness. Unlike conventional tasks such as simple button pressing, the CAT cannot be performed successfully unless the subject is paying close attention to the task. Subjects were free to start a work-set at any time after the preceding reward was finished, given that an ensuing alcohol reward would not rise BrAC above the 180 mg% safety limit. They chose whether they would next work for alcohol or for saline, by using the respective button labeled “A” for alcohol or “W” for water. Subjects could take as much time as they wished to complete the work-set, including pausing or ceasing work altogether. Once a work-set was completed, subjects were informed that the reward was now being delivered and when it ended so that another work set could begin. According to a progressive-ratio schedule, the number of correct CAT trials required to complete a work-set increased exponentially. The progression was identical for alcohol and saline, but accounted separately. The time required to complete a work-set typically lasted about 30 s for the first and about 23 min for the 15th work-set. Participants were only very vaguely informed about the increasing workload and did not know how many correct trials they had do perform for the current work-set, or how much time this might take. Table S1 in the supplement shows the progression of correct CAT trials required to complete the work-sets after starting the VWR phase.

### DNA preparation and genotyping

Genomic DNA was isolated from blood using the QIAamp DNA micro kit (Qiagen, Germany) according to the manufacturer’s protocol. The OPRM1 A118G SNP (rs1799971) was detected by a TaqMan SNP Genotyping Assay (C_8950074_1; Applied Biosystems, Carlsbad, California) on an ABI 7900 HT RT‐PCR system with SDS 2.2.2 software (10 μl reaction volume containing 10 ng genomic DNA, 40 cycles of 95 °C for 15 s, and 60 °C for 1 min).

Naltrexone and 6ß-Naltrexol blood levels were quantified by chromatography and mass spectrometry. Methodological details are provided in the supplement.

### Data analysis

All data were entered and stored in a MACRO database, then exported to and analyzed in IBM SPSS 25 for Windows. The validated database system MACRO was set up in compliance with GCP and the requirements of privacy, the role/rights principle and includes an audit trail. The database was programmed, validated and administered by the Coordinating Centre for Clinical Trials and was hosted on the servers of the Technische Universität Dresden. Missing, questionable, or additional information was requested on a regular basis by the risk-based central and onsite monitoring. The primary endpoint was the difference in cWFA in the CAT (in the naltrexone and placebo group) between session 1 and session 2. Secondary outcome variables were the cumulative work for saline (cWFS), peak and mean BrAC at sessions 1 and 2. The normal distribution of the data was verified by Shapiro–Wilk test. For normally distributed data, the evaluation was performed using two-sided t-tests or Pearson correlation coefficients. For non-normally distributed data, two-sided Mann-Whitney tests or Spearman correlation coefficients were used. One subject in the naltrexone group withdrew consent before the second session. For analyses of the primary outcome in the ITT population, we imputed the missing data by taking his baseline cWFA and subtracting the mean change in cWFA of the other *n* = 21 subjects in the naltrexone group.

Additional information on methods according to the Consolidated Standards of Reporting Trials is provided in the supplement.

## Results

The study medication (naltrexone and placebo) and experimental procedures were generally well tolerated. One participant in the naltrexone group withdrew because of fatigue and hair loss attributed to the study medication. There was one severe adverse event (wasp sting in the throat), unrelated to the experimental interventions. Sample characteristics are summarized in Table 1. cWFA and cWFS in the placebo group correlated significantly between the two ASA sessions (Pearson’s *r* = 0.636, *p* < 0.001 and *r* = 0.743, *p* < 0.001, respectively), suggesting good test-retest stability.

### Analyses of the full experimental time

**Fig. 2:**
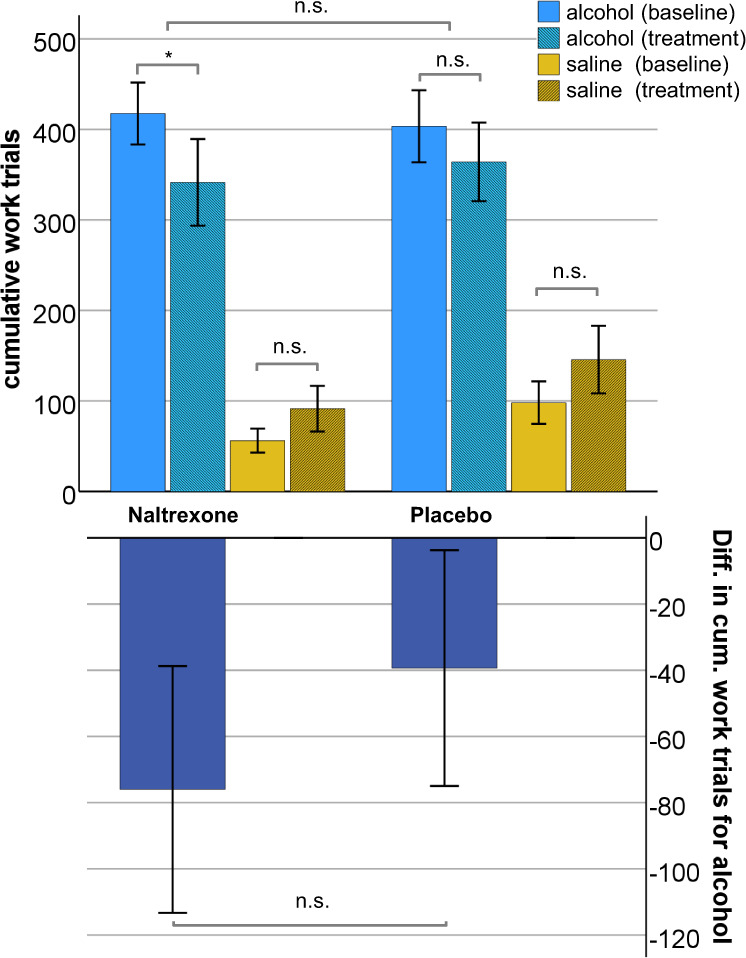
Treatment outcomes. Cumulative work for alcohol and for saline before and during treatment in the ITT sample (upper panel) and primary study outcome (lower panel) *p < 0.05; ***p < 0.001.

The cumulative work for alcohol (cWFA) and for saline (cWFS) in the intention-to-treat (ITT) sample is depicted in Fig. 2.

Participants worked significantly more for alcohol than for saline in both groups, before and during treatment (*p* < 0.01, respectively). Naltrexone, but not placebo treatment, decreased cWFA significantly compared to the respective pretreatment baseline. Analyzing the difference of cWFA as our primary outcome parameter, we found that the group difference in reducing WFA was not statistically significant (−76 ± 174.5 for naltrexone vs. −39 ± 166.7 for placebo, *t* (44) = 0.73, *p* = 0.471). The effect size for the naltrexone effect, according to Cohen, was *d* = 0.215 (95% CI −0.367–0.794).

The mean sum and standard deviation (SD) of naltrexone and beta-naltrexol blood levels in the naltrexone group on the second session were 55.4 ± 27.6 ng/ml and were significantly related to naltrexone-induced changes of cWFA (*r* = −0.53; *p* = 0.014), peak BAC (*r* = −0.45; *p* = 0.034) and mean BAC (*r* = −0.52; *p* = 0.016). See Figs. S1–S3 in the supplement for scatter plots depicting these interrelations. Comparing the treatment effect between the eight OPRM1 G-allele carriers and the 14 AA participants in the naltrexone group, we found no significant difference (−123 ± 170 AG/GG vs. −49 ± 165 AA, *t*(20) = 1.0; *p* = 0.327).

Analyzing secondary outcome measures we found that, compared to placebo, naltrexone treatment did not significantly affect cWFS (see Fig. 2) or the changes in peak BrAC, mean BrAC, OCDS craving scores, or TLFB measures of real-life drinking (see Table 1). Naltrexone blood levels were not significantly related to changes in any of these measures.

**Table 1:** Subject characteristics and secondary outcome variables in the ITT (intention-to-treat) sample.

	Group	Data were expressed as mean ± SD or no. (%)			Placebo vs. naltrexone baseline	Placebo vs. naltrexone Diff. treatment - baseline	
	Placebo		Naltrexone				cohens *d* (95% CI)
**Demographics**							
Participants (males/females)	**24 (20/4)**		**22 (20/2)**		*χ*²(1) = 0.581; *p* = 0.446		
Age (yrs, mean ± SD) [min–max]	29, 8 ± 5,5 (25–50)		29, 4 + 6,2 (25–54)		*t*(44) = 0.243; *p* = 0.809		
Regular smokers (%)	14 (58.3)		16 (72.7)		*χ*²(1) = 1.048; *p* = 0.306		
OPRM1-G carriers (%)	9 (37.5)		8 (36.4)		*χ*²(1) = 0.006; *p* = 0.936		
Family history of alcoholism (%)	4 (16.7)		3 (13.6)		*χ*²(1) = 0.082; *p* = 0.775		
Education					*χ*²(4) = 3.712; *p* = 0.446		
No vocational training (%)	3 (12.5)		0				
Vocational training ongoing (%)	2 (8.3)		2 (9.1)				
Completed vocational training (%)	7 (29.2)		5 (22.7)				
Ongoing university education (%)	8 (33.3)		9 (40.9)				
University degree (%)	4 (16.7)		6 (27.3)				
**Baseline** **clinical symptom ratings**							
Alcohol dependence scale	5.87 ± 2.94		7.45 ± 4.17		*t*(43) = −1.478; *p* = 0.147		
AUDIT	13.29 ± 3.928		14.36 ± 3.723		*t*(44) = −0.948; *p* = 0.348		
**Secondary outcome measures**	Baseline	During treatment	Baseline	During treatment			
OCDS-score	8.8 ± 3.9	7.3 ± 4.1*	9.1 ± 4.3	8.8 ± 4.3	*t*(44) = −0.291; *p* = 0.772	*t*(38) = −0.310; *p* = 0.759	*d* = −0.099 (−0.726–0.529)
% drinking days	69.7 ± 15.1	63.5 ± 20.2	73.7 ± 15.5	67.0 ± 20.8*	*t*(44) = −0.894; *p* = 0.376	*t*(44) = 0.101; *p* = 0.920	*d* = 0.030 (−0.549–0.608)
% binge days	47.5 ± 16.2	41.3 ± 18.7	47.1 ± 16.6	39.6 ± 18.0*	*t*(44) = 0.088; *p* = 0.93	*t*(44) = 0.281; *p* = 0.78	*d* = 0.083 (−0.496–0.661)
Grams alcohol/drinking day	109.7 ± 40.8	105.3 ± 40.8	107.7 ± 43.4	101.7 ± 49.5	*t*(44) = 0.168; *p* = 0.867	*t*(43) = 0.176; *p* = 0.861	*d* = 0.053 (−0.534–0.638)
% CDT	1.67 ± 1.76	2.03 ± 2.29	2.30 ± 3.60	2.14 ± 3.49	*t*(44) = −0.757; *p* = 0.453	*t*(44) = 1.838; *p* = 0.073	*d* = 0.543 (−0.050–1.129)
GGT (µmol/s*l)	0.60 ± 0.35	0.59 ± 0.33	0.75 ± 0.55	0.71 ± 0.73	*t*(44) = −1.130; *p* = 0.265	*t*(44) = 0.488; *p* = 0.628	*d* = 0.144 (−0.436–0.722)
ALAT (µmol/s*l)	0.55 ± 0.31	0.5 ± 0.28	0.55 ± 0.18	0.53 ± 0.17	*t*(44) = −0.042; *p* = 0.967	*t*(44) = −0.397; *p* = 0.693	*d* = −0.117 (−0.696–0.262)
ASAT (µmol/s*l)	0.48 ± 0.13	0.46 ± 0.15	0.48 ± 0.12	0.46 ± 1.13	*t*(44) = 0.144; *p* = 0.886	*t*(44) = −0.219; *p* = 0.828	*d* = −0.065 (−0.643–0.515)
Peak BrAC (mg%)	114 ± 22	111 ± 27	119 ± 25	107 ± 33	*t*(44) = −0.88; *p* = 0.384	*t*(44)=1.287; *p* = 0.205	*d* = 0.380 (−0.206–0.962)
Mean BrAC (mg%)	91 ± 21	87 ± 24	96 ± 21	85 ± 29	*t*(44) = −0.86; *p* = 0.395	*t*(43) = 1.020; *p* = 0.313	*d* = 0.305 (−0.286–0.892)
Cumulative work for saline	98.13 ± 113.84	145.71 ± 182.94	60.91 ± 63.28	96.14 ± 115.03	*t*(44) = 1.344; *p* = 0.177	*t*(44) = 0.361; *p* = 0.720	*d* = 0.106 (−0.473–0.685)

All particiapants were of caucasian ethnicity.

*ALAT* alanine aminotransferase, *ASAT* aspartate aminotransferase, *AUDIT* alcohol use disorders identification test, *BrAC* breath alcohol concentration, *CDT* carbohydrate-deficient Transferrin, *OCDS* obsessive compulsive drinking scale, *OPRM-1* opioid receptor Mu 1.

*Significantly different from baseline at *p* < 0.05.

### Analysis by low- and high-demand ASA interval

**Fig. 3:**
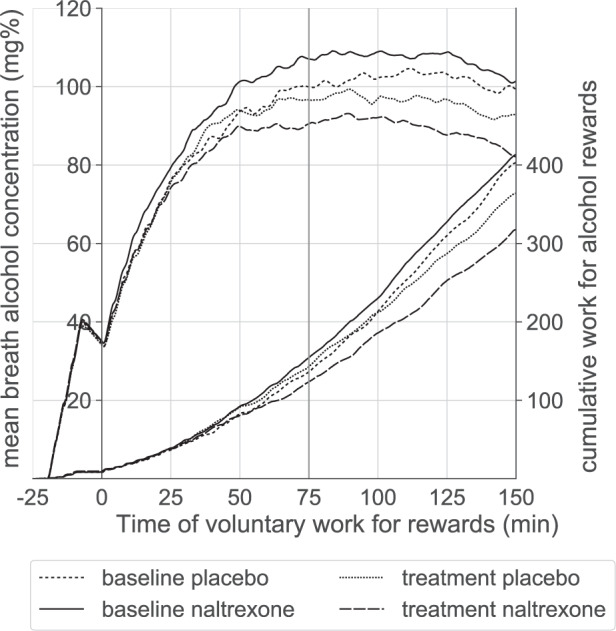
Mean BrAC and cWFA over experimental time. Time −25 until 0 min = priming interval with experimenter-prompted work. Voluntary work for rewards started at time zero. Time 0–75 min = ascending phase; time 75–150 min = plateau phase.

As noted earlier, the experiment was designed such that, for subjects working consistently for alcohol, the BrAC trajectory would ascend quickly in the beginning, then level off, merge into a plateau and even decline as a function of the work schedule (see Fig. S4). Figure 3 demonstrates that on average, this transition does occur in both groups, before and during treatment, with BrACs plateauing approximately between minutes 75 and 125 of the VWR phase.

We, therefore, explored whether naltrexone treatment would affect VWR differently during the first and the second half of the experiment. We based these analyses on a predefined per-protocol sample, excluding three subjects of the naltrexone group: one who did not complete both sessions and two naltrexone group subjects in whom the second-session naltrexone blood levels were zero. Due to this hypothesis-generating approach, no adjustment was applied for multiple testing.

**Fig. 4:**
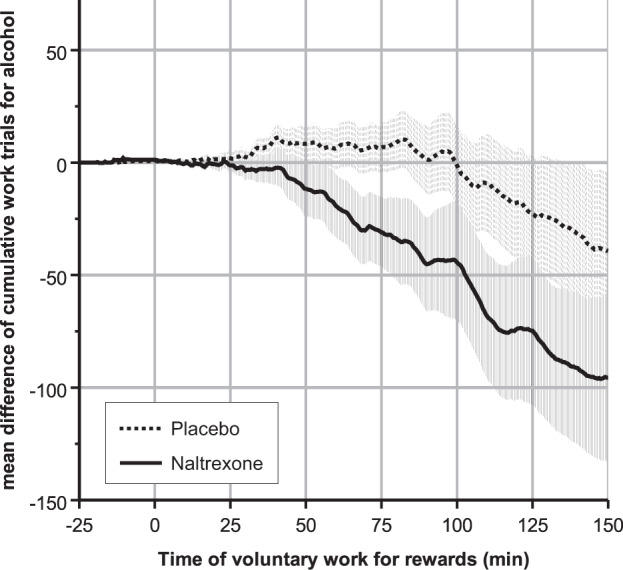
Medication effect on cumulative work for alcohol over experimental time. Medication effect (difference between treatment and baseline) on cumulative work for alcohol over experimental time. Shaded areas = SEM. Note: 0–75 min = ascending phase of BrAC; 75–150 min = plateau phase of BrAC.

During the initial ascending phase (start to 75 min of VWR), cumulative work for alcohol decreased in the naltrexone and slightly increased in the placebo group (mean difference in cWFA: −32 ± 67.2 vs. + 6 ± 53.1, t(41 )= 2.093, *p* = 0.043), Cohen’s *d* = 0.643 (95% CI 0.021–1.257, see Fig. 4). During the plateau phase, however, the group difference in change of cWFA was not statistically significant (−64.7 ± 119.4 for naltrexone vs. −46 ± 143.1 for placebo, *t*(41) = 0.457, *p* = 0.65), Cohen’s *d* = 0.14 (95% CI −0.450–0.758).

Treatment-induced changes of the secondary outcome variables cWFS, peak BrAC, or mean BrAC did not differ between groups, neither during the ascending nor the plateau phase.

Analyses of the correlations between naltrexone blood levels and treatment effects for both phases were performed in the ITT sample in order to include the two participants with zero blood levels. Higher naltrexone blood levels were associated with a more pronounced decrease in cWFA (*r* = −0.49, *p* = 0.021 for the ascending phase and *r* = −0.44, *p* = 0.04 for the plateau phase).

### Analyses of subjective measures

Baseline subjective measures are presented in Table S2 and did not significantly differ between ASA sessions for each treatment group, nor between groups (all *p* > 0.2). Spearman’s rho correlation coefficient was used to assess the relationship between cWFA and changes in subjective responses, defined as slopes between time points 2 to 3 and 3 to 4 (see Table 2). During the ascending phase of first ASA, cWFA was significantly and positively associated with changes in stimulation and wellbeing, but not for alcohol “desire”; while in the plateau phase, cWFA was only and positively associated with changes in “desire”. The same pattern was observed during the second ASA session for the placebo group. In the naltrexone group, the associations with stimulation and wellbeing changes were lost, while an association with “desire” occurred during the ascending, but not the plateau phase.

**Table 2:** Spearman’s rho correlation coefficients between cumulative work for alcohol (cWFA) and slopes of subjective responses in the ascending and plateau phases of the first and second alcohol self-administration (ASA) session in the intention-to-treat (ITT) sample.

		cWFA 1st ASA				cWFA 2nd ASA	
		Ascending phase		Plateau phase		Ascending phase	Plateau phase
Stimulation	Placebo	0.211 *p* = 0.323	**0.374**^**a**^ ***p*** **=** **0.010**	0.129 *p* = 0.558	0.186 *p* = 0.232	**0.444**^**b**^ ***p*** **=** **0.03**	−0.118 *p* = 0.593
	Naltrexone	**0.437**^**b**^ ***p*** **=** **0.042**		0.218045 *p* = 0.356		−0.008 *p* = 0.973	0.085 *p* = 0.716
Wellbeing	Placebo	0.337 *p* = 0.108	**0.328**^**b**^ ***p*** **=** **0.026**	−0.032 *p* = 0.886	0.180 *p* = 0.248	**0.449**^**b**^ ***p*** **=** **0.028**	0.33 *p* = 0.124
	Naltrexone	0.358 *p* = 0.102		**0.447**^**b**^ ***p*** **=** **0.048**		0.092 *p* = 0.691	0.010 *p* = 0.964
Alcohol desire	Placebo	0.006 *p* = 0.979	0.094 *p* = 0.531	0.233 *p* = 0.284	**0.380**^**b**^ ***p*** **=** **0.012**	0.286 *p* = 0.175	**0.582**^**a**^ ***p*** **=** **0.004**
	Naltrexone	0.137 *p* = 0.545		**0.588**^**a**^ ***p*** **=** **0.006**		**0.54**^**b**^ ***p*** **=** **0.012**	0.193 *p* = 0.402

^a^Significant correlation at *p* < 0.01.

^b^Significant correlation at *p* < 0.05.

The bold values indicate significance.

## Discussion

Regarding the primary endpoint of this clinical trial, we were unable to demonstrate a statistically significant overall effect of naltrexone on cWFA. However, in exploratory analyses separating the first (“ascending”) and the second (“plateau”) half of the VWR time span, we found that naltrexone significantly reduced cWFA in the former, but not the latter.

The null finding concerning the predefined primary endpoint can be best explained by the observation that naltrexone did not affect WFA during the plateau phase of the experiment. Reasons to explain this are discussed below. By design of the study, the major proportion of work was performed during the plateau phase (see Fig. 3), which therefore contributed numerically more to cWFA than the ascending phase. Alternatively, while naltrexone reduced cWFA almost twice as much as the placebo, our sample may not have been sufficiently powered to detect a small-moderate naltrexone effect.

We were surprised by how strongly the naltrexone effect on WFA depended on the low- versus high-demand ASA interval. We suggest interpreting this result with respect to the differing reward contingencies and incentive components driving WFA. First, we discuss results of the ascending phase, where we assumed that WFA would be positively reinforced such that work rapidly increases BrAC, this exposure resulting in pleasant subjective perceptions, which then promote further work (see introduction). Our experimental control was confined to the first of these three elements, preventing conclusions on causal relations between the others. However, the finding that cWFA was positively associated with concurrent changes in subjective stimulation and wellbeing is at least compatible with the notion that positive consequences of alcohol exposure gained some control over behavior, as in positive reinforcement. Interestingly, the same association was also observed during the second session in subjects threated with placebo, but not in the naltrexone group, suggesting that naltrexone-treated participants might have worked less for alcohol because of attenuated positive reinforcement. Such an interpretation is in line with previous studies describing that naltrexone reduces the rewarding feelings caused by alcohol in social drinkers [30, 31].

Our finding of a statistically significant medication effect during the ascending phase is noteworthy, since these results were obtained with a parallel-group design in a relatively small sample of nondependent subjects. Naltrexone’s effect on low-demand cWFA was significantly related to its blood levels, supporting a conclusion that that the attenuation of WFA was truly caused by the drug. Nine previous laboratory studies investigated naltrexone’s effect on free-access ASA [17]; out of those, only O’Malley et al. [32]. reported a larger effect size than Cohen’s *d* of 0.64 observed by us. However, design differences (consumption vs. work) and route of administration (oral vs. intravenous) make it difficult to compare these studies in more detail. There is; however, one naltrexone study that used a progressive-ratio VWR design which resembled ours, but employed simple keyboard button presses as a work task and administered the alcohol rewards orally [33]. No effect of naltrexone on cWFA was observed in 40 subjects using a cross-over design in that study. We conclude that the ascending phase of the TESMA paradigm is more sensitive to detect a naltrexone effect than other laboratory ASA paradigms.

The observation that naltrexone treatment altered only laboratory behavior, but not the subjects’ real-life drinking, alcohol craving, or blood parameters of alcohol consumption is an ambiguous finding. Since naltrexone undoubtfully helps to reduce drinking, this could mean that the TESMA, designed as a screening tool, actually is more sensitive than mere clinical observation, detecting a real drug effect already in a small sample within a short time. Our sample may have also been motivated to reduce their drinking despite their non-treatment-seeking status. Participants reduced their average grams of alcohol per drinking day, percent drinking days, and percent binge days as a function of treatment with both naltrexone and placebo. The reduction from baseline associated with naltrexone treatment reached significance for percent drinking days and binge days (Table 1). On the other hand, it remains possible that the TESMA might feign a false positive drug effect. Consequently, our findings should be interpreted carefully and warrant replication in a sample where a drug significantly changes ASA both in the TESMA and in real life.

Discussing reasons why a naltrexone effect was not detected during the high-demand ASA phase is more difficult. From a statistical point of view, the reason might simply be that the SEM of the outcome variable steadily increased over time, averaging ~25 at the end of the ascending phase and 75 towards the end of the experiment, while its group difference remained largely unchanged throughout the plateau phase (see Fig. 4). Several unspecific determinants of human behavior can be assumed to have gained influence over the passing of experimental time, such as getting bored or starved, being distracted by missing online connection to social networks, or thinking about the next activities after leaving the lab. To prevent such an effect, future lab studies could be shortened. Since naltrexone blood levels and the naltrexone effect on WFA were significantly interrelated when analyzing only the plateau phase, we think that naltrexone still exerted some effect, irrespective of the experimental conditions.

Two more conceptual interpretations of the results during the high-demand ASA phase refer again to the reward contingencies and incentive components driving WFA. As outlined in the introduction, we assume that WFA during that period was negatively reinforced. This notion is supported by scorings of subjective alcohol “desire”, which can be interpreted as dissatisfaction with the perceived alcohol effects. “Desire” slopes correlated positively with WFA during the plateau-, but not during the ascending phase, which implies that subjects used WFA to fight unpleasant feelings caused by falling BrAC, i.e., negative reinforcement. In this light, the absent naltrexone effect might match recent findings in clinical trials. While naltrexone reduces heavy drinking in alcohol-dependent patients [16], this might, in fact, only be true if drinking is driven by positive reinforcement, such as seeking euphoria. Several studies could not detect a naltrexone effect in persons who use alcohol primarily for relief from unpleasant affective states [34–36]. The common feature linking the plateau phase in social drinkers and relief drinking in alcoholics is that drinking is not driven by positive reinforcement, implying that the TESMA might have reproduced these clinical observations.

Yet another interpretation of the high-demand phase refers to the motivation to work. Continuing to work for alcohol despite long periods without getting rewarded can be interpreted as an expression of alcohol “wanting.” This pathological motivation is thought to specifically characterize subjects with substance dependence [37]. Since we included only nondependent participants, their “wanting” must be expected to be rather low, maybe too low to provide enough bandwidth for a naltrexone effect to be observed (a floor-effect).

While naltrexone treatment affected cWFA, it did not change the peak or mean BrAC.

To make the above-described differences in reward contingencies possible, we critically depended on the use of i.v. administration. While increasing BrACs to induce positive reinforcement can be easily achieved by oral administration, obtaining experimental control over falling BrAC is much harder. With the TESMA, the infusion rate is adapted immediately after each reward in order to decrease BrAC at a steady slope of −0.8 mg/dL per minute; nearly four times faster than the ~12–15 mg/dL per hour observed after oral ingestion. BrAC is closely related to arterial blood concentration [38] and thus brain alcohol exposure [39], and we previously reported that moderate drinkers felt more “intoxicated” and more “high” during ascending vs. descending BrAC limbs of a comparable infusion experiment [40].

Regarding the A118G polymorphism of the OPRM1 gene, G-allele carriers were slightly, but not statistically significantly, more responsive to naltrexone treatment than AA subjects, which is in line with a prior study employing free-access i.v. ASA [41]. Nevertheless, we think that our effort to enrich the study population with G-allele carriers probably helped to demonstrate a naltrexone effect.

Some fundamental methodological goals of the TESMA paradigm were also accomplished. We demonstrated test-retest reliability for cWFA and cWFS. The fact that cWFA was significantly greater than cWFS indicates that, by design, alcohol was the “primary” reward and that the subjects’ effort was not driven by nonspecific reasons such as boredom or thirst. Their work resulted in substantial alcohol exposure with peak BrAC before treatment averaging 117 ± 23 mg/dL, and 91 % of all subjects reaching binge levels above 80 mg/dL. This peak exposure compares well with that reported by previous studies using i.v. alcohol administration [11, 12, 29, 42]. Alcohol exposure was substantially higher than with the published oral ASA studies, offering space for treatment-induced reductions and allaying concerns about a floor-effect.

A specific strength of our study is that it was registered with the European Medicines Agency, rigorously complied with all regulations of good clinical practice as defined by the international conference of harmonization of (GCP-ICH) and still was finished in a single center within a relatively short time of 1 year and ten months. The mandatory results reporting was uploaded at the EMA website [43].

Limitations included that, due to ethical concerns, we could not include alcohol-dependent subjects, which may partly explain why we observed only ambiguous effects of naltrexone treatment on real-life drinking. Second, females were grossly underrepresented, requiring further studies to examine gender-specific effects in our paradigm. Third, highly educated individuals were overrepresented; therefore, it remains unclear whether our results can be generalized to the general population. In addition, working for alcohol precluded more frequent measurements of subjective effects, which would have been desirable to strengthen our conclusions on drinking motivations.

We conclude that the TESMA is a safe and practical method that has the potential to enable quick and efficient screening of new drugs for their efficacy to attenuate alcohol consumption under conditions of positive reinforcement of drinking. It probably also provides a condition of negative reinforcement, and for the first time, provides experimental evidence suggesting that naltrexone’s effect might depend on reward contingency. This result, however, requires confirmation by studies where timing and progressive-ratio work schedules are specifically devised to investigate negative reinforcement, and which include alcohol-dependent patients.

## Supplementary information

Supplemental Material

## References

[1] GBD. (2018). 2016 alcohol collaborators. Alcohol use and burden for 195 countries and territories, 1990-2016: a systematic analysis for the Global Burden of Disease Study 2016. Lancet.

[2] Jonas DE, Amick HR, Feltner C, Bobashev G, Thomas K, Wines R (2014). Pharmacotherapy for adults with alcohol use disorders in outpatient settings: a systematic review and meta-analysis. JAMA..

[3] Skinner MD, Lahmek P, Pham H, Aubin HJ (2014). Disulfiram efficacy in the treatment of alcohol dependence: a meta-analysis. PLoS ONE.

[4] Ray LA, Bujarski S, Roche DJO, Magill M (2018). Overcoming the “Valley of Death” in medications development for alcohol use disorder. Alcohol Clin Exp Res.

[5] Litten RZ, Egli M, Heilig M, Cui C, Fertig JB, Ryan ML (2012). Medications development to treat alcohol dependence: a vision for the next decade. Addict Biol.

[6] Kranzler HR, Soyka M (2018). Diagnosis and pharmacotherapy of alcohol use disorder: a review. JAMA..

[7] Yardley MM, Ray LA (2017). Medications development for the treatment of alcohol use disorder: insights into the predictive value of animal and human laboratory models. Addict Biol.

[8] Zimmermann US, O’Connor S, Ramchandani VA (2013). Modeling alcohol self-administration in the human laboratory. Curr Top Behav Neurosci.

[9] Zimmermann US, Mick I, Laucht M, Vitvitskiy V, Plawecki MH, Mann KF (2009). Offspring of parents with an alcohol use disorder prefer higher levels of brain alcohol exposure in experiments involving computer-assisted self-infusion of ethanol (CASE). Psychopharmacology.

[10] Barrett SP, Tichauer M, Leyton M, Pihl RO (2006). Nicotine increases alcohol self-administration in non-dependent male smokers. Drug Alcohol Depend.

[11] Plawecki MH, Wetherill L, Vitvitskiy V, Kosobud A, Zimmermann US, Edenberg HJ (2013). Voluntary intravenous self-administration of alcohol detects an interaction between GABAergic manipulation and GABRG1 polymorphism genotype: a pilot study. Alcohol Clin Exp Res.

[12] Farokhnia M, Grodin EN, Lee MR, Oot EN, Blackburn AN, Stangl BL (2018). Exogenous ghrelin administration increases alcohol self-administration and modulates brain functional activity in heavy-drinking alcohol-dependent individuals. Mol Psychiatry.

[13] Robinson TE, Berridge KC (2001). Incentive-sensitization and addiction. Addiction..

[14] Cyders MA, Plawecki MH, Corbin W, King A, McCarthy DM, Ramchandani VA (2020). To Infuse or Ingest in Human Laboratory Alcohol Research. Alcohol Clin Exp Res.

[15] Plawecki MH, Durrani AM, Boes J, Wetherill L, Kosobud A, O’Connor S (2019). Comparison of subjective responses to oral and intravenous alcohol administration under similar systemic exposures. Alcohol Clin Exp Res.

[16] Rösner S, Hackl‐Herrwerth A, Leucht S, Vecchi S, Srisurapanont M, Soyka M. Opioid antagonists for alcohol dependence. Cochrane Database Syst Rev. 2010;12:CD001867. 10.1002/14651858.CD001867.pub3 PMC13590218 21154349

[17] Hendershot CS, Wardell JD, Samokhvalov AV, Rehm J (2017). Effects of naltrexone on alcohol self-administration and craving: meta-analysis of human laboratory studies. Addict Biol.

[18] Fang X, Deza-Araujo YI, Petzold J, Spreer M, Riedel P, Marxen M, et al. Effects of moderate alcohol levels on default mode network connectivity in heavy drinkers. Alcohol Clin Exp Res. 2021;45:1039–50. 10.1111/acer.14602 33742481

[19] Riedel P, Wolff M, Spreer M, Petzold J, Plawecki MH, Goschke T (2021). Acute alcohol does not impair attentional inhibition as measured with Stroop interference scores but impairs Stroop performance. Psychopharmacology.

[20] Sobell L, Sobell M. Timeline follow-back. Humana Press; 1992.

[21] Rice JP, Reich T, Bucholz KK, Neuman RJ, Fishman R, Rochberg N (1995). Comparison of direct interview and family history diagnoses of alcohol dependence. Alcohol Clin Exp Res.

[22] Gelernter J, Kranzler H, Cubells J (1999). Genetics of two mu opioid receptor gene (OPRM1) exon I polymorphisms: population studies, and allele frequencies in alcohol- and drug-dependent subjects. Mol Psychiatry.

[23] Ramchandani VA, Umhau J, Pavon FJ, Ruiz-Velasco V, Margas W, Sun H (2011). A genetic determinant of the striatal dopamine response to alcohol in men. Mol Psychiatry.

[24] Bilbao A, Robinson JE, Heilig M, Malanga CJ, Spanagel R, Sommer WH (2015). A pharmacogenetic determinant of mu-opioid receptor antagonist effects on alcohol reward and consumption: evidence from humanized mice. Biol Psychiatry.

[25] Chamorro AJ, Marcos M, Miron-Canelo JA, Pastor I, Gonzalez-Sarmiento R, Laso FJ (2012). Association of micro-opioid receptor (OPRM1) gene polymorphism with response to naltrexone in alcohol dependence: a systematic review and meta-analysis. Addict Biol.

[26] Ray LA, Chin PF, Miotto K (2010). Naltrexone for the treatment of alcoholism: clinical findings, mechanisms of action, and pharmacogenetics. CNS Neurol Disord Drug Targets.

[27] Bach P, Vollsta Dt-Klein S, Kirsch M, Hoffmann S, Jorde A, Frank J (2015). Increased mesolimbic cue-reactivity in carriers of the mu-opioid-receptor gene OPRM1 A118G polymorphism predicts drinking outcome: a functional imaging study in alcohol dependent subjects. Eur Neuropsychopharmacol.

[28] Jünger E, Gan G, Mick I, Seipt C, Markovic A, Sommer C (2016). Adolescent women induce lower blood alcohol levels than men in a laboratory alcohol self-administration experiment. Alcohol Clin Exp Res.

[29] Plawecki MH, White K, Kosobud AEK, Grahame N, Zimmermann US, Crabb D (2018). Sex differences in motivation to self-administer alcohol after 2 weeks of abstinence in young-adult heavy drinkers. Alcohol Clin Exp Res.

[30] Ray LA, Hutchison KE (2007). Effects of naltrexone on alcohol sensitivity and genetic moderators of medication response: a double-blind placebo-controlled study. Arch Gen Psychiatry.

[31] Drobes DJ, Anton RF, Thomas SE, Voronin K (2004). Effects of naltrexone and nalmefene on subjective response to alcohol among non-treatment-seeking alcoholics and social drinkers. Alcohol Clin Exp Res.

[32] O’Malley SS, Krishnan-Sarin S, Farren C, Sinha R, Kreek MJ (2002). Naltrexone decreases craving and alcohol self-administration in alcohol-dependent subjects and activates the hypothalamo-pituitary-adrenocortical axis. Psychopharmacology.

[33] Setiawan E, Pihl RO, Cox SM, Gianoulakis C, Palmour RM, Benkelfat C (2011). The effect of naltrexone on alcohol’s stimulant properties and self-administration behavior in social drinkers: influence of gender and genotype. Alcohol Clin Exp Res.

[34] Mann K, Roos CR, Hoffmann S, Nakovics H, Leménager T, Heinz A (2018). Precision medicine in alcohol dependence: a controlled trial testing pharmacotherapy response among reward and relief drinking phenotypes. Neuropsychopharmacology..

[35] Witkiewitz K, Roos CR, Mann K, Kranzler HR (2019). Advancing precision medicine for alcohol use disorder: replication and extension of reward drinking as a predictor of naltrexone response. Alcohol Clin Exp Res.

[36] Roos CR, Bold KW, Witkiewitz K, Leeman RF, DeMartini KS, Fucito LM, et al. Reward drinking and naltrexone treatment response among young adult heavy drinkers. Addiction. 2021;116:2360–71. 10.1111/add.15453 PMC8328878 33620746

[37] Berridge KC, Robinson TE (2016). Liking, wanting, and the incentive-sensitization theory of addiction. Am Psychol.

[38] Lindberg L, Brauer S, Wollmer P, Goldberg L, Jones AW, Olsson SG. Breath alcohol concentration determined with a new analyzer using free exhalation predicts almost precisely the arterial blood alcohol concentration. Forensic Sci Int. 2007;168:200–7. 10.1016/j.forsciint.2006.07.018 16978819

[39] Gomez R, Behar KL, Watzl J, Weinzimer SA, Gulanski B, Sanacora G (2012). Intravenous ethanol infusion decreases human cortical γ-aminobutyric acid and N-acetylaspartate as measured with proton magnetic resonance spectroscopy at 4 tesla. Biol Psychiatry.

[40] Wetherill L, Morzorati SL, Foroud T, Windisch K, Darlington T, Zimmerman US (2012). Subjective perceptions associated with the ascending and descending slopes of breath alcohol exposure vary with recent drinking history. Alcohol Clin Exp Res.

[41] Sloan ME, Klepp TD, Gowin JL, Swan JE, Sun H, Stangl BL (2018). The OPRM1 A118G polymorphism: converging evidence against associations with alcohol sensitivity and consumption. Neuropsychopharmacology..

[42] Bujarski S, Jentsch JD, Roche DJO, Ramchandani VA, Miotto K, Ray LA (2018). Differences in the subjective and motivational properties of alcohol across alcohol use severity: application of a novel translational human laboratory paradigm. Neuropsychopharmacology..

[43] European Medicines Agency. Clinical trial results: validation of a test system for development of medications for alcoholism. https://www.clinicaltrialsregister.eu/ctr-search/trial/2015-002831-16/results (2021).

