# Supplementary material for: Using naltrexone to validate a human laboratory test system to screen new medications for alcoholism (TESMA)- a randomized clinical trial
Source: Transl Psychiatry. 2023 Apr 5;13:113. doi: 10.1038/s41398-023-02404-7 (PMC10076427; doi:10.1038/s41398-023-02404-7)
Supplement: Supplementary file 1 — Supplemental Material [file 41398_2023_2404_MOESM1_ESM.pdf]

# Using naltrexone to validate a human laboratory test system to screen new medications for alcoholism (TESMA)- a randomized clinical trial

# Supplementary information

## Naltrexone and 6ß-Naltrexol quantification with mass spectrometry

Sample preparation: serum (100 µL) was mixed with 20 µL internal standard in acetonitrile (50 ng/mL naltrexone-D3, 50 ng/mL 6-ß-naltrexol-D3) and 200 µL 0.2 M $ZnSO_4$. The mixture was vortexed for 30 s and then centrifuged for 5 min at 2000 x g. The supernatant was diluted 1:3 with mobile phase A and 10 µL were injected into the chromatographic system. The 10 point serum calibration ranged from 1.0 to 250 ng/mL for both compounds.

Chromatography and mass spectrometry: chromatographic separation was conducted on an Acquity UPLC system (Waters, Eschborn) using a 2.1 x 150 mm, 1.8 µm, HSS T3 column (Waters, Eschborn) maintained at 40°C. Mobile phase A consisted of 20 mM ammonium formate, 0.1% formic acid (pH 3), and mobile phase B consisted of 0.1% formic acid in methanol. Elution was conducted with a linear gradient spanning 2.5 min at a flow rate of 0.35 mL/min beginning at 95% A , 5% B, and ending at 5% A, 95% B. Data were acquired on a Xevo TQ-MS detector (Waters, Eschborn) with an ESI source operating in positive ionization, MRM mode. The following transitions were monitored for target ions and qualifier ions 1 and 2: Naltrexone: 342.1 > 267.1, 342.1 > 282.0, 342.1 > 324.1 ; Naltrexone-D3: 345.1 > 270.2, 345.1 > 285.1, 345.1 > 327.1 ; 6ß-Naltrexol: 344.2 > 254.1, 344.2 > 272.1, 344.2 > 308.2 ; 6ß-Naltrexol-D3: 347.2 > 254.2, 347.2 > 272.2, 347.2 > 311.2. At least 15 data points per peak were recorded at a dwell time of 30 ms. The instrument settings for the ion source were: capillary voltage: 1.5 kV, cone voltage: 40 kV, desolvation temperature: 650°C, source temperature: 150°C, desolvation gas flow: 1000 L/h, cone gas flow: 50 L/h, collision gas (argon) flow: 0.20 mL/min.

## Urine screening for substance abuse

The Nal von Minden Drug screen Multi 10R tested for amphetamines, barbiturates, benzodiazepines, cocaine, MDMA, methamphetamine, morphine/ opiates, tricyclic antidepressants, THC and tramadol.

## Supplementary figures and tables

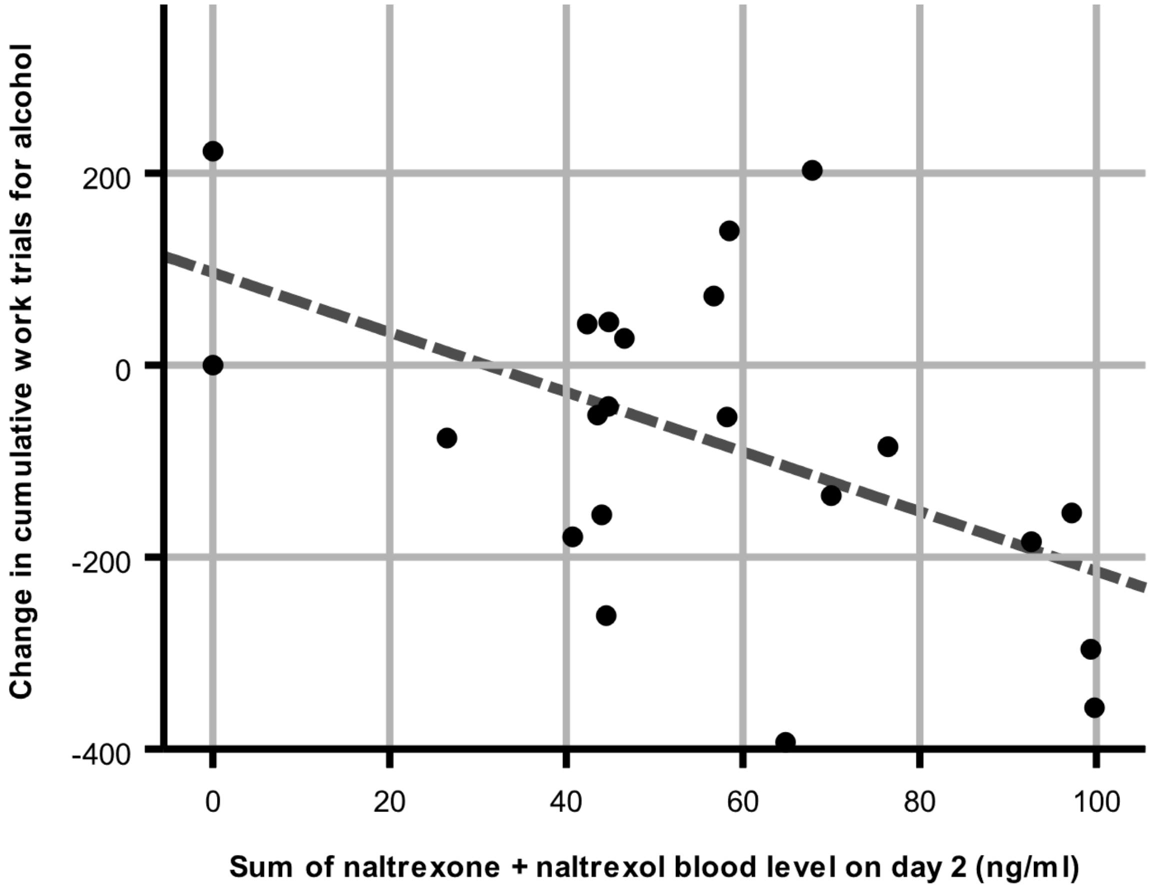


**Fig. S1:** Difference between treatment and baseline in cumulative work trials for alcohol vs. sum of naltrexone and naltrexol blood levels (ng/ml) on session 2.

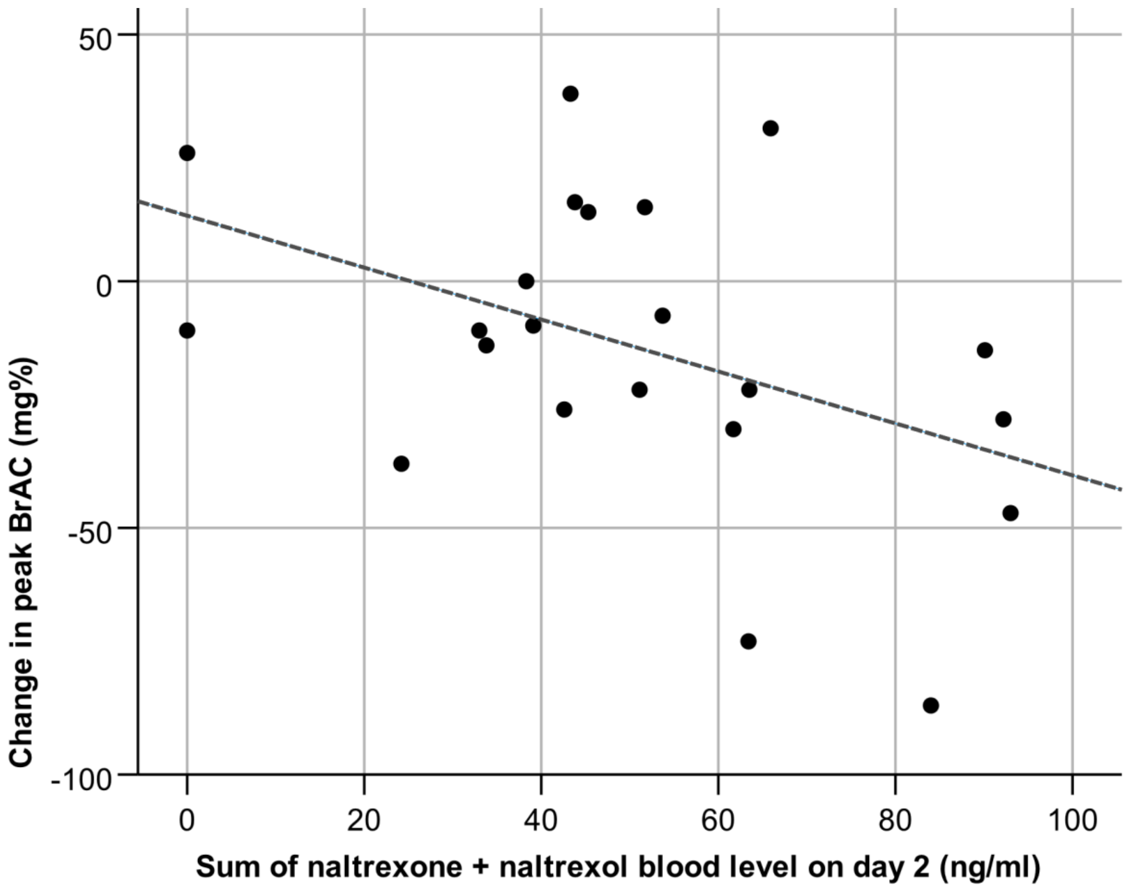


**Fig. S2:** Difference between treatment and baseline in peak breath alcohol concentration (mg %) vs. sum of naltrexone and naltrexol blood levels (ng/ml) on session 2.

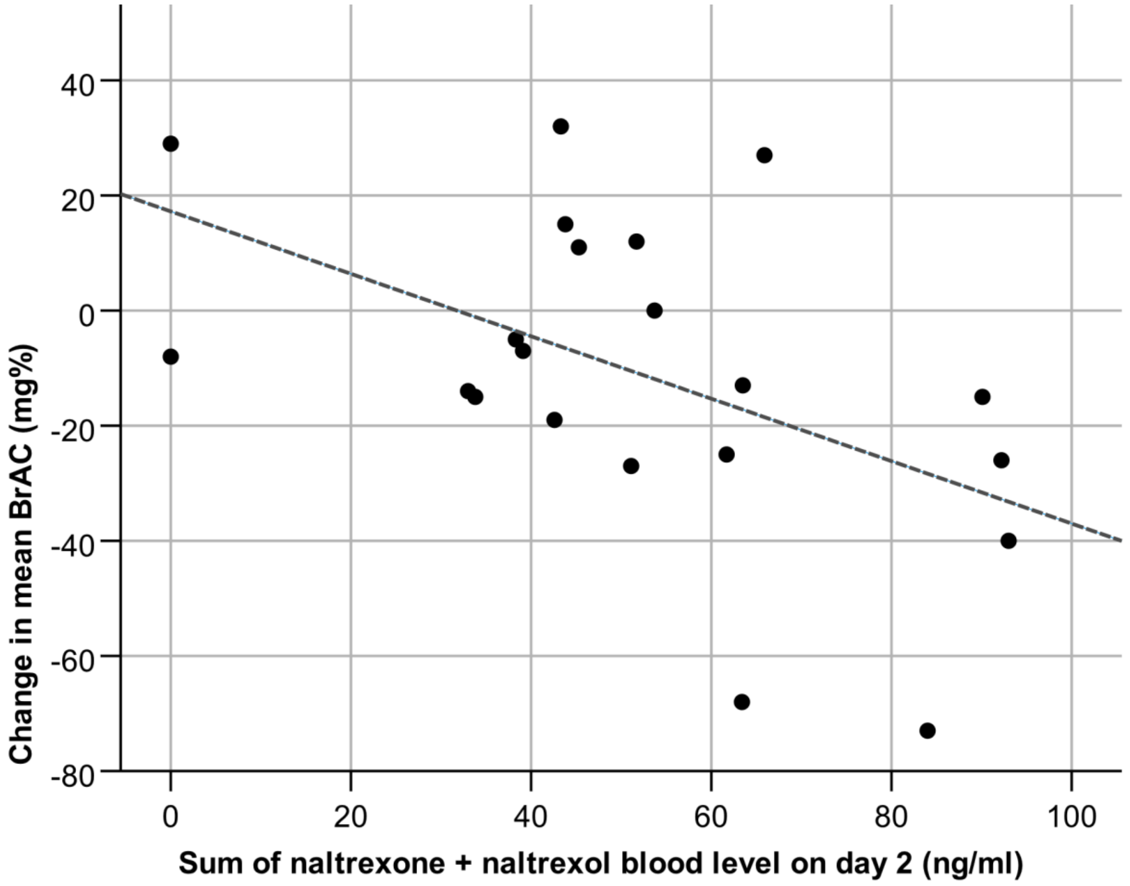


**Fig. S3:** Difference between treatment and baseline in mean breath alcohol concentration (mg %) vs. sum of naltrexone and naltrexol blood levels (ng/ml) on session 2.

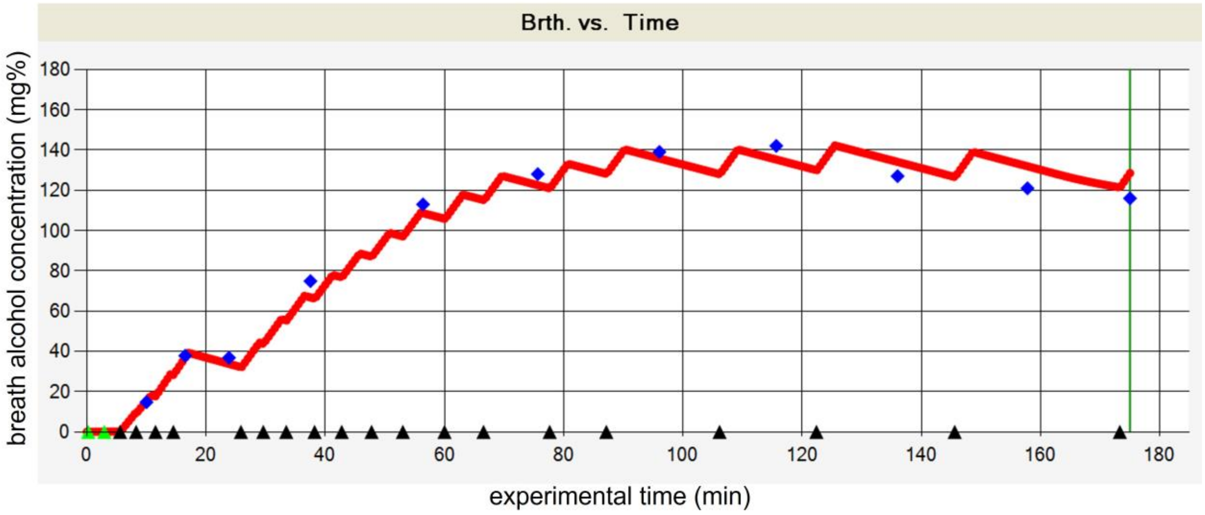


**Fig. S4:** Example of an individual experiment depicting breath alcohol (mg%) over experimental time (min). Black triangles: alcohol rewards delivered; green triangles: saline rewards delivered, red line: BrAC trajectory as modelled by the CAIS system, blue dots: actual breath alcohol readings

| CAT work set | Number of correct CAT trials during voluntary work for rewards |
|---|---|
| 1 | 2 |
| 2 | 2 |
| 3 | 3 |
| 4 | 4 |
| 5 | 5 |
| 6 | 7 |
| 7 | 9 |
| 8 | 12 |
| 9 | 16 |
| 10 | 22 |
| 11 | 29 |
| 12 | 39 |
| 13 | 53 |
| 14 | 72 |
| 15 | 99 |

**Tab. S1:** Number of correct CAT trials required to complete consecutive work sets

| | **Group** | | | | **placebo vs. naltrexone (before treatment)** |
|---|---|---|---|---|---|
| | **Placebo** | | **Naltrexone** | | |
| | 1st ASA (before treatment) | 2nd ASA (during treatment) | 1st ASA (before treatment) | 2nd ASA (during treatment) | |
| **stimulation** | 44.521 ± 25.512 | 48.467 ± 48.467 | 41.057 ± 28.808 | 35.119 ± 28.957 | t(43)=0.428; p=0.671 |
| **sedation** | 35.926 ± 35.925 | 45.617 ± 45.617 | 29.186 ± 31.903 | 31.895 ± 31.896 | t(33.403)=-0.852; p=0.413 |
| **negative effects** | 2.179 ± 8.767 | 0.888 ± 0.888 | 5.886 ± 15.123 | 11.224 ± 23.171 | t(43)=-1.021; p=0.313 |
| **alcohol desire** | 19.033 ± 20.22 | 22.792 ± 22.792 | 20.776 ± 23.351 | 22.367 ± 23.232 | t(43)=-0.268; p=0.790 |
| **wellbeing** | 77.525 ± 18.600 | 73.271 ± 73.271 | 70.743 ± 19.023 | 64.776 ± 21.749 | t(43)=1.207; p=0.234 |
| **drunkeness** | 0.263 ± 0.747 | 0.258 ± 0.258 | 0.695 ± 1.592 | 0.21 ± 0.833 | t(43)=-1.192; p=0.240 |
| **thirst** | 40.266 ± 31.945 | 40.217 ± 40.217 | 42.714 ± 29.397 | 36.305 ± 28.001 | t(43)=-0.266; p=0.791 |

**Tab. S2:** subjective measures at baseline (min 0) in the ITT sample (100 mm visual analog scales). Data are expressed as mean ± SD.

## Information according to CONSORT Statement

| Section/Topic | Item No | Checklist item | Reported on page No |
|---|---|---|---|
| **Title and abstract** | | | |
| | 1a | Identification as a randomized trial in the title | Article, p. 1 |
| | 1b | Structured summary of trial design, methods, results, and conclusions | Article, p. 2 (Abstract) |
| **Introduction** | | | |
| Background and objectives | 2a | Scientific background and explanation of rationale | Article, pp. 3-4 (Introduction) |
| | 2b | Specific objectives or hypotheses | Article, p 4,<br>Supplement, p. 11 |
| **Methods** | | | |
| Trial design | 3a | Description of trial design (such as parallel, factorial) including allocation ratio | Article, pp. 4-6 (Methods) |
| | 3b | Important changes to methods after trial commencement (such as eligibility criteria), with reasons | Supplement, p. 12 |
| Participants | 4a | Eligibility criteria for participants | Article, p. 5<br>Supplement, pp. 12-13 |
| | 4b | Settings and locations where the data were collected | Supplement, p. 13 |
| Interventions | 5 | The interventions for each group with sufficient details to allow replication, including how and when they were actually administered | Article, pp. 5-7 |
| Outcomes | 6a | Completely defined pre-specified primary and secondary outcome measures, including how and when they were assessed | Article, p 7,<br>Supplement, p. 14 |
| | 6b | Any changes to trial outcomes after the trial commenced, with reasons | not applicable |
| Sample size | 7a | How sample size was determined | Supplement, p. 14 |
| | 7b | When applicable, explanation of any interim analyses and stopping guidelines | Supplement, p. 15 |

| | | | |
|---|---|---|---|
| **Randomisation** | | | |
| Sequence generation | 8a | Method used to generate the random allocation sequence | Supplement, p. 16 |
| | 8b | Type of randomisation; details of any restriction (such as blocking and block size | Supplement, p. 16 |
| Allocation concealment mechanism | 9 | Mechanism used to implement the random allocation sequence (such as sequentially numbered containers), describing any steps taken to conceal the sequence until interventions were assigned | Supplement, p. 16 |
| Implementation | 10 | Who generated the random allocation sequence , who enrolled participants, and who assigned participants to interventions | Supplement, p. 16 |
| Blinding | 11a | If done, who was blinded after assignment to interventions (for example, participants, care providers, those assessing outcomes) and how | Supplement, p. 16 |
| | 11b | If relevant, description of the similarity of interventions | Supplement, p. 16 |
| Statistical methods | 12a | Statistical methods used to compare groups for primary and secondary outcomes | Tab. 1 (Patient Characteristics)<br>Article pp. 4-6 |
| | 12b | Methods for additional analyses, such as subgroup analyses and adjusted analyses | Article pp. 4-6 |
| **Results** | | | |
| Participant flow (a diagram is strongly recommended) | 13a | For each group, the numbers of participants who were randomly assigned, received intended treatment, and were analysed for the primary outcome | Fig. 1<br>Consort Flow Chart |
| | 13b | For each group, losses and exclusions after randomisation, together with reasons | Fig. 1<br>Consort Flow Chart |
| Recruitment | 14a | Dates defining the periods of recruitment and follow-up | Supplement, p. 17 |
| | 14b | Why the trial ended or was stopped | Supplement, p. 17 |
| Baseline data | 15 | A table showing baseline demographic and clinical characteristics for each group | Tab. 1<br>Patient Characteristics |
| Numbers | 16 | For each group, number of participants (denominator) included in each analysis and | Fig. 1 |

| | | | |
|---|---|---|---|
| analysed | | whether the analysis was by original assigned groups | Consort Flow Chart |
| Outcomes and estimation | 17a | For each primary and secondary outcome, results for each group, and the estimated effect size and its precision (such as 95% confidence interval) | Article, pp. 7-8<br>Supplement, p. 17 |
| | 17b | For binary outcomes, presentation of both absolute and relative effect sizes is recommended | not applicaple |
| Ancillary analyses | 18 | Results of any other analyses performed, including subgroup analyses and adjusted analyses, distinguishing prespecified from exploratory | Article, pp. 7-8 |
| Harms | 19 | All important harms or unintended effects in each group | Supplement, p. 17 |
| **Discussion** | | | |
| Limitations | 20 | Trial limitations, addressing sources of potential bias, imprecision, and, if relevant, multiplicity of analyses | Article p. 9-11<br>(Discussion) |
| Generalisability | 21 | Generalisability (external validity, applicability) of the trial findings | Article p. 9-11 (Discussion) |
| Interpretation | 22 | Interpretation consistent with results, balancing benefits and harms, and considering other relevant evidence | Article p. 9-11 (Discussion) |
| **Other information** | | | |
| Registration | 23 | Registration number and name of trial registry | Article p. 4 (Introduction) |
| Protocol | 24 | Where the full trial protocol can be accessed, if available | Supplement, p. 17 |
| Funding | 25 | Sources of funding and other support (such as supply of drugs), role of funders | Article p. 12 (Funding) |

Table S3 | CONSORT 2010 checklist of information to include when reporting a randomised trial

# Supplementary objectives (CONSORT Checklist item 2b)

The following is a list of the complete objectives of the clinical trial. In the present paper, only the highlighted objectives (*) are addressed. For a complete overview, please refer to results reporting or Riedel et al. [1] and Fang et al. [2]

main objective:

General Objective of the study was to demonstrate that a laboratory alcohol self-administration method can predict the therapeutic potential of new compounds to reduce relapse in alcohol-dependent patients. The opiate antagonist Naltrexone, whose anti-relapse effect is well documented, was used as a reference substance for validation. The main objective was, that naltrexone administration compared to placebo reduces the willingness to perform work for alcohol (WFA), which was operationalized as the number of work trials in a constant attention task (CAT)*.

Secondary objectives

- administration of naltrexone in comparison to placebo leads to a reduction of alcohol craving and real-life drinking*
- administration of naltrexone in comparison to placebo leads to reduction of the CDT-level*
- administration of naltrexone in comparison to placebo leads to a change in perception of subjective alcohol effects
- the effectiveness of naltrexone can be predicted by the A118G polymorphism of the OPRM1*
- administration of naltrexone changes the baseline and alcohol-induced ability of motor inhibition (see Riedel et al. [1])
- administration of naltrexone changes the baseline and alcohol-induced regional cerebral perfusion and the the baseline and alcohol-induced cerebral resting state activity (see Fang et al. [2])
- changes of alcohol effects to the brain activity induced by naltrexone in comparison to placebo correlate with effects of naltrexone on the willingness to work for alcohol self-administration

# Supplementary Methods

## Important changes to methods after trial commencement (CONSORT checklist item 3b)

The initial exclusion criterion "reported alcohol consumption within the 3 days before screening, measured with Timeline Follow-back Interview and negative ETG" was discarded, because several subjects had negative ETG values, although they had consumed a small amount of alcohol during this period.

The initial exclusion criterion “body weight > 95 kg” was changed to “body weight > 100 kg” and later on to “body weight > 130 kg”. The reason was that, contrary to our initial assumption, infusion rates sufficient to achieve the targeted BrAC values could also be achieved in subjects with a body weight of up to 130 kg.

A re-screening was implemented: Before trial commencement the maximum time lag allowed between screening and session 1 was defined as 42 days. This timeframe could not be observed several times, due to delays in OPRM1 genotyping. If 42 days between screening and session 1 were exceeded, the inclusion and exclusion criteria were reconfirmed in a re-screening.

Adjustment of the times in the visit schedule was necessary, because the initially strict schedules could not be adhered by several subjects due to time constraints.

One secondary outcome measure was more precisely defined, to avoid divergent interpretations: “Drinking habits, measured with Timeline Follow-Back Interview, during the 45 days prior to study entry (measured at screening or re-screening), and throughout the entire duration of intake of the investigational drug.” (The first definition comprised the entire study duration from screening, not the entire duration of intake of the investigational drug.)

Further minor changes affecting only visits 3 and 4 are not mentioned here.

## Comprehensive eligibility criteria for participants (CONSORT checklist item 4a)

Inclusion criteria:

- Male or female volunteers between 25 and 55 years of age
- at least weekly alcohol consumption at a medium risk level according to WHO, measured with Timeline Follow-back Interview over the last 45 day with an average amount of alcohol of 41 g/day (men) or 31 g/day (women)
- at least 6 days with an alcohol consumption of >100 g/day (men) or 75 g/day (women) and at least 4 non consecutive alcohol abstinent days in the last 45 days
- at least 1 drinking day in each full week between screening and visit 1 and not more than 6 abstinent days in the week before visit 1
- no wish for changing risky alcohol consumption
- Written informed consent

exclusion criteria:

- a history of hypersensitivity against alcohol or one of the used medicinal products, of their ingredients or medicinal products with similar chemical structures

- participation in another clinical trial within the last 4 weeks before inclusion
- addiction or other disorders, which will not allow the subject to assess the character and importance or possible consequences of the clinical trial
- pregnant or breastfeeding women
- women capable of bearing children, except women who fulfil following criteria:- post-menopausal (12 months natural amenorrhoea or 6 month amenorrhoea and Serum FSH >40 ml U/ml) - post operative (6 weeks after bilateral ovarectomy with or without hysterectomy) - regular and correct use of a contraceptive method with an error Quote of < 1 % per year (for example implants, depot injections, oral contraceptive, IUP). It has to be recognized that a combined oral contraception - in contrast to pure progesterone compounds - have a failure rate of < 1 %. Hormone IUDs with a Pearl Index of 1 % are safer than copper IUDs. - sexual abstinence - vasectomy of the Partner
- evidence that the participant is not expected to comply with the protocol (for example lacking compliance)
- current or previous alcohol or substance dependence according to DSM-IV (exception: tobacco dependence)
- current or previous treatment for alcohol-realted problems, for example addiction treatment services, self-help group, detoxification treatment
- current or previous diseases, for which alcohol infusion could cause a clinically relevant hazard (e. g. pancreatitis, liver cirrhosis)
- current or planned intake of opiate analgesics
- current psychiatric treatment or intake of psychiatric drug, or suffering from of a psychiatric disease which should be treated
- a history of suicide attempts
- Clinical Institute Withdrawal od Alcohol (CIWA) scale -score >5 at screening
- a history of symptoms of alcohol withdrawal, epileptic seizures or delirium
- routine laboratory parameters indicating relevant liver-, pancreas- or kidney injury, an acute infection, anaemia or lack of vitamins (ASAT, ALAT > twofold elevated at screening, gamma-GT, lipase >threefold elevated, CRP < 15 mg/l, creatin indicating at least moderate renal insufficiency ( eGFR <60 ml/min), leucocytes > 12000/µl, haemoglobin < 7,5 mmol/l (men) or 6,5 mmol/l (women), MCV > 100 fl)
- Body weight > 130 kg
- urine drug screening: : positive at screening visit for opiates, cannabinoids, cocaine, amphetamines, benzodiazepines. Or positive at visit 1 for opiates. Or positive for cannabis, cocaine, amphetamines, benzodiazepines at the first and a second try to perform visit 1
- breath alcohol concentration above zero at the screening visit or on the first and a second try to perform visit 1
- unfit for fMRT (e. g. cardiac pacemaker, claustrophobia)
- specific contraindications against naltrexone: acute hepatitis, severe or acute liver disease, severe kidney disease, rare hereditary galactose intolerance, Lapp-lactase-deficiency or Glucose-galactose-malabsorption,

## Settings and locations where the data were collected (CONSORT checklist item 4b)

All data were collected at the „Klinik und Poliklinik für Psychiatrie und Psychotherapie“, University Hospital Carl Gustav Carus, Dresden, Germany.

## Comprehensive outcome measures (CONSORT checklist item 6a)

Primary end point was the difference of cumulative number of work sets for alcohol in the CAT (in the naltrexone and placebo group) between 1st and 2nd ASA session (session 1 and session 2).

Timepoints of evaluation: session 1: 7 - 42 days after Screening (or re-screening). Session 2: 7 - 10 days after session 1.

Secondary end points:

(1) Endpoints concerning efficiency

- Difference between ASA session 1 (visit 1) and session 2 (visit 2) of the "break point" in the "progressive work" schedule for the work for alcohol
- Maximum blood alcohol concentration during alcohol self-administration at session 1 and 2
- Difference between session 1 and session 2 of the cumulative number of work sets for saline in the CAT
- Drinking habits measured with Timeline Follow-back Interview over 45 days before screening (measured at screening or re-screening) and over the entire duration of intake of the medicinal product (day 1 until last day of intake of medicinal product, measured at the follow up)
- CDT – level: (carbohydrate-deficient transferrin), measured at session and follow up
- Alcohol craving in daily routine (OCD – scale) measured at visit 1 and visit 4
- Difference in subjective alcohol effects between session 1 and 2, measured with visual analogue scales ("Quizzer") before, during and after the infusion
- Capacity for motor impulse control during infusion of physiologic saline solution or alcohol (single-blinded), measured with the counting stroop task at visit 3 and 4
- Regional cerebral perfusion during infusion of sodium chloride solution or alcohol (single-blinded), measured with arterial spin labeling (ASL) under verum or placebo condition at visit 3 and 4.
- Cerebral resting state activity during infusion of saline solution or alcohol (single-blinded), measured with BOLD fMRI under verum or placebo condition at visit 3 and 4

(2) Endpoints concerning safety during the treatment

- Laboratory tests: ALAT, ASAT, gamma-GT, creatinine, lipase, CRP, standard blood cell count before inclusion (screening visit); ALAT, ASAT, standard blood cell count at visit 4 and ALAT, ASAT, standard blood cell count, lipase and gamma-GT after finishing all study relating interventions (visit 5)
- Medical survey concerning occurring adverse events at visit 1 to 5

## Sample size (CONSORT checklist item 7a)

Due to the novelty of our design, we could not base our sample size determination on data generated with the same experimental setup. However, our primary outcome measure – the

difference between baseline and treatment in cumulative work for alcohol (cWFA) in the constant attention task (CAT) – had already been investigated under different conditions [3].

In previous alcohol self-administration experiments, subjects reduced and decelerated their alcohol consumption after administration of naltrexone, resulting in a lower BrAC [5,6], which corresponded to findings from clinical trials that naltrexone reduced the number of drinks per drinking day [4].

Additionally, it had been shown, that work for alcohol in a progressive work schedule under experimental conditions is related to real-world alcohol consumption outside of a laboratory setting [7].

Based on these data and our prior experience with free-access alcohol self-administration (ASA), we simulated drinking patterns for 30 experimental participants (15 in a naltrexone condition, 15 in a placebo condition) on 2 experimental ASA  sessions. We simulated that the average maximum BrAC was similar in both groups on the first ASA session, did not chance on the 2nd ASA session for the placebo group, but was 10 % lower on the 2nd session in the naltrexone group. This corresponded to the average naltrexone-induced reduction in drinking volume per drinking day in clinical trials (10.83 percent according to Rösner et al. [4].

In a next step, we applied these simulated data to the TESMA progressive work paradigm, determining which amount of WFA would have been required to reach the simulated alcohol exposure for each subject. The result was a mean decrease in  cWFA of 130 in the naltrexone group and 30 in the placebo group (standard deviation 100).

Using the "nQuery Advisor" software, a test significance level of $\alpha$=0.05 and a power of 80% yielded a case number of 17 participants per treatment arm. Assuming a drop-out rate of 8 participants per treatment arm, the number of cases per treatment arm was set at 25.

## Interim analyses and stopping guidelines (CONSORT checklist item 7b)

An official interim analysis was not planned.

An independent Data Safety Monitoring Board (DSMB), two external experts and one biometrician, regularly evaluated safety aspects of the clinical trial with regard to the occurrence of serious adverse events and the following stopping criteria. The sponsor was entitled to terminate the clinical trial prematurely in case of:

- Insufficient recruitment rate
- serious problems with data quality
- unexpected circumstances at the trial site that make it impossible to continue the clinical trial
- early evidence of superiority or inferiority of a treatment group (in an independent interim analysis)
- unacceptable risks and toxicities
- new scientific evidence that did not allow the continuation of the trial.
- more than one naltrexone-associated suicide attempt or naltrexone-associated rhabdomyolysis
- increase of daily alcohol consumption by an average of 50 percent during participation in the trial (compared to the period before participation) in an independent interim analysis of the first 25 completed subjects

None of the aforementioned criteria had to be applied.

## Supplemental Randomization Information (CONSORT checklist items 8-10)

A computer assisted randomization with a block size of 4 was performed. The random allocation sequence was generated with the software nQuery Advisor ®6.01. Strata were (1) sex (male/female); G-Allele-carriers of the A118G polymorphism of the OPRM1 (yes/no); and smoking status (yes/ no).

The allocation to the treatment groups was carried out according to a randomization list based on the consecutively assigned subject numbers. The randomization list was created in an access-protected manner. It was only visible to the independent biometrician and the employee who entered it into the electronic database (eCRF).

The random allocation sequence was generated by a biometrician. An authorized blinded investigator enrolled the participants. According to the randomization result, participants were assigned to interventions by the blinded investigators.

## Blinding (CONSORT checklist items 11a)

Regarding drug treatment allocation, the study was triple blinded.  Study participants, care providers (study nurses, study physicians etc.), investigators and all persons who analyzed the data did not know which subject was randomized into the naltrexone and the placebo group.

Regarding alcohol self-administration, the study was single-blinded. Participants did not know the amount of alcohol already administered. Experimenters did know the subjects' BrAC at any time, in order to ensure their safety.

## Investigational medicinal products and similarity (CONSORT checklist items 11b)

The capsules of the investigational medicinal products were identical in appearance, color, and weight in both groups.

The blinded naltrexone hydrochloride 25 mg and 50 mg capsules as well as the matching placebo capsules were manufactured and packaged under GMP conditions using validated processes.

For storage at room temperature (15 to 25 °C), a shelf life of 12 months was demonstrated in the selected primary packaging material.

The quality control performed due to European Pharmacopeia confirmed that the contents of the capsules containing the active ingredients was in accordance with the specifications and that there were no degradation products or impurities.

The randomization of the blinded investigational medicinal products was carried out in a ratio of 1:1 according to the randomization list before handover to the investigator.

# Supplementary results

## Periods of recruitment and follow-up (CONSORT checklist items 14a, 14b)

The recruitment period started in November 2015 and ended in July 2017.

First patient first visit date was December 11, 2015, last patient last visit date September 4, 2017.

The study was discontinued when expiration date of the investigational medical products had been reached. Sample size had been determined at 17 per treatment arm plus an assumed drop out rate of 8 per treatment arm. Since drop out rate was lower than anticipated and calculated sample size was reached, the study ended prematurely with an actual enrollment of 46 participants (see CONSORT flow chart, Fig. 1).

## Important harms or unintended effects (CONSORT checklist item 19)

In summary, the investigational drug was well tolerated and can be considered safe.

The total number of participants with adverse events was higher in the naltrexone than in the placebo group, mainly due to the more frequent occurrence of fatigue and nausea.

2 subjects decided to discontinue the study medication and further participation in study-related interventions. One subject reported persistent joint pain. The other reported subjectively perceived hair loss. Dose changes were not possible due to the medication regimen. Due to the low side effects analyzing specific groups with increased risk was not done.

During the study one SAE – a short-term hospitalization triggered by a wasp sting, for which no association with the study medication can be discerned occurred. The subject concerned had been assigned to the naltrexone group.

# Additional study information

The full trial protocol is not available online. On request, we are happy to provide the complete protocol in German language, as submitted to the competent authority (German BfArM) and the Ethics Committee.

Sponsor's Protocol Code Number: TUD-TEMANX-065. Ethical review committee’s code: EK 413092015.

# Supplement References


1 Riedel P, Wolff M, Spreer M, Petzold J, Plawecki MH, Goschke T, et al. Acute alcohol does not impair attentional inhibition as measured with Stroop interference scores but impairs Stroop performance. Psychopharmacology (Berl). 2021;238(6):1593-607.

2 Fang X, Deza-Araujo YI, Petzold J, Spreer M, Riedel P, Marxen M, et al. Effects of moderate alcohol levels on default mode network connectivity in heavy drinkers. Alcohol Clin Exp Res. 2021.

3 Plawecki MH, Wetherill L, Vitvitskiy V, Kosobud A, Zimmermann US, Edenberg HJ, et al. Voluntary intravenous self-administration of alcohol detects an interaction between GABAergic manipulation and GABRG1 polymorphism genotype: a pilot study. Alcohol Clin Exp Res. 2013;37 Suppl 1:E152-60.

4 Rösner S, Hackl-Herrwerth A, Leucht S, Vecchi S, Srisurapanont M, Soyka M. Opioid antagonists for alcohol dependence. 2010.

5 Drobes DJ, Anton RF, Thomas SE, Voronin K. A clinical laboratory paradigm for evaluating medication effects on alcohol consumption: naltrexone and nalmefene. Neuropsychopharmacology. 2003;28(4):755-64.

6 O'Malley SS, Krishnan-Sarin S, Farren C, Sinha R, Kreek MJ. Naltrexone decreases craving and alcohol self-administration in alcohol-dependent subjects and activates the hypothalamo-pituitary-adrenocortical axis. Psychopharmacology (Berl). 2002;160(1):19-29.

7 Setiawan E, Pihl RO, Cox SM, Gianoulakis C, Palmour RM, Benkelfat C, et al. The effect of naltrexone on alcohol's stimulant properties and self-administration behavior in social drinkers: influence of gender and genotype. Alcohol Clin Exp Res. 2011;35(6):1134-41.
